# A Regulatory Loop of FBXW7-MYC-PLK1 Controls Tumorigenesis of MYC-Driven Medulloblastoma

**DOI:** 10.3390/cancers13030387

**Published:** 2021-01-21

**Authors:** Dong Wang, Angela Pierce, Bethany Veo, Susan Fosmire, Etienne Danis, Andrew Donson, Sujatha Venkataraman, Rajeev Vibhakar

**Affiliations:** 1 Department of Pediatrics, Anschutz Medical Campus, University of Colorado, Aurora, CO 80045, USA; dong.2.wang@cuanschutz.edu (D.W.); ANGELA.PIERCE@CUANSCHUTZ.EDU (A.P.); BETHANY.VEO@CUANSCHUTZ.EDU (B.V.); susan.fosmire@cuanschutz.edu (S.F.); ETIENNE.DANIS@CUANSCHUTZ.EDU (E.D.); ANDREW.DONSON@CUANSCHUTZ.EDU (A.D.); SUJATHA.VENKATARAMAN@CUANSCHUTZ.EDU (S.V.); 2 Morgan Adams Foundation Pediatric Brain Tumor Research Program, Children’s Hospital Colorado, Aurora, CO 80045, USA; 3 Department of Neurosurgery, University of Colorado Denver, Aurora, CO 80045, USA

**Keywords:** FBXW7, MYC, PLK1 inhibition, medulloblastoma

## Abstract

**Simple Summary:**

Group 3 medulloblastoma (MB) is often accompanied by *MYC* amplification and has a poor prognosis. FBXW7, a critical tumor suppressor in many types of cancer, regulates the proteasome-mediated degradation of oncoproteins including MYC. However, the role of FBXW7 in the tumorigenesis of group 3 MB has not been well studied. In this study, we show that *FBXW7* is downregulated in group 3 MB patient samples, and FBXW7 stabilization is crucial for inhibiting c-MYC. We identified a FBXW7-MYC-PLK1 regulatory loop in MYC-driven MB, which provides a mechanism of using protein kinase inhibitors for translation in the future.

**Abstract:**

Polo-like kinase 1 (*PLK1*) is highly expressed in group 3 medulloblastoma (MB), and it has been preclinically validated as a cancer therapeutic target in medulloblastoma. Here, we demonstrate that PLK1 inhibition with PCM-075 or BI6727 significantly reduces the growth of MB cells and causes a decrease of *c-MYC* mRNA and protein levels. We show that MYC activates *PLK1* transcription, while the inhibition of PLK1 suppresses MB tumor development and causes a decrease in c-MYC protein level by suppressing FBXW7 auto poly-ubiquitination. FBXW7 physically interacts with PLK1 and c-MYC, facilitating their protein degradation by promoting ubiquitination. These results demonstrate a PLK1-FBXW7-MYC regulatory loop in MYC-driven medulloblastoma. Moreover, FBXW7 is significantly downregulated in group 3 patient samples. The overexpression of *FBXW7* induced apoptosis and suppressed proliferation in vitro and in vivo, while constitutive phosphorylation mutation attenuated its tumor suppressor function. Altogether, these findings demonstrated that PLK1 inhibition stabilizes FBXW7 in MYC-driven MB, thus revealing an important function of *FBXW7* in suppressing medulloblastoma progression.

## 1. Introduction

Brain tumors are the most common cause of oncological death in children, and medulloblastoma (MB) is a malignant childhood brain tumor, accounting for 20–25% of pediatric brain tumors [1,2,3]. Recent genomic analyses have identified multiple sub-groups with differing outcomes, underscoring the heterogeneity of MB [4]. By current international consensus, there are four main sub-groups of MB: WNT, SHH, group 3, and group 4 with multiple subtypes [5,6]. Group 3 patients, in particular, express high levels of *c-MYC* and have the worst prognosis [7]. Thus, there is a critical need for more effective and targeted therapies for group 3 MB.

Polo-like kinases (PLKs) comprise a family of five serine/threonine protein kinases [8]. The best characterized PLK family member is *PLK1*, which regulates cell-cycle progression by mediating various steps during mitosis [9]. The inhibition of PLK1 prevents cell proliferation, self-renewal, cell-cycle progression, and induced apoptosis [10,11]. We and others have previously identified *PLK1* as a key regulator of medulloblastoma cell viability [12,13]. Despite considerable study, it is not yet clear why the expression of *PLK1* is upregulated and how a high level of *PLK1* reprograms cells to promote the cancer state in medulloblastoma.

The *MYC* family of transcription factors are known to impact proliferation, survival, and metabolism in the development of cancer, including MB [14]. Though MYC inhibition would be a powerful approach for the treatment of many types of cancers, the direct targeting of MYC has been a challenge for decades due to its “undruggable” protein structure [15]. Hence, alternatives to an MYC blockade have been widely explored to achieve desirable anti-tumor effects, including MYC/MAX complex disruption, *MYC* transcription, translation inhibition, and MYC destabilization [16].

FBXW7 is a critical tumor suppressor and one of the most commonly deregulated ubiquitin–proteasome system proteins in human cancer [17,18,19]. It is a component of the SCF-like ubiquitin ligase complex that targets MYC for proteasomal degradation. The downregulation of FBXW7 leads to the synergistic accumulation of cellular and active chromatin-bound MYC in various types of cancer [20]. FBXW7 controls the proteasome-mediated degradation of oncoproteins such as Cyclin E, c-MYC, MCL-1, mTOR, JUN, NOTCH-1, and AURKA [21]. However, the mechanisms by which FBXW7 modulates the tumorigenesis of MB is not well delineated.

Here, we demonstrate that PLK1 promotes FBXW7 auto poly-ubiquitination and proteasomal degradation, counteracting the FBXW7-mediated degradation of c-MYC in MB cells. In turn, stabilized c-MYC directly activates *PLK1* transcription, constituting a regulatory loop. FBXW7 acts as a tumor suppressor in MYC-amplified medulloblastoma: the overexpression of *FBXW7* induces cell apoptosis, suppresses cell proliferation, and improves the survival of orthotopic xenograft bearing mice. Together, our results reveal a PLK1-FBXW7-MYC signaling circuit that underlies tumor pathogenesis and provide a potential strategy for the activation of FBXW7 against c-MYC-driven MB.

## 2. Results

### 2.1. MYC Activates PLK1 Transcription in MYC-Amplified Medulloblastoma Cell Lines

**Figure 1:**
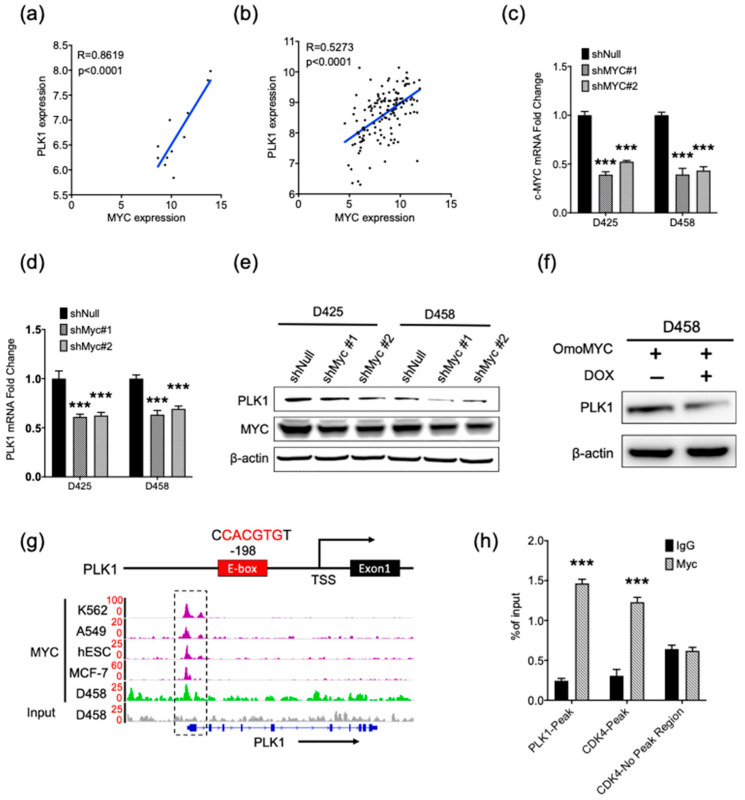
MYC activates polo-like kinase 1 (*PLK1*) transcription in MYC-amplified medulloblastoma (MB) cell lines. (**a**) Correlation of mRNA levels between of *PLK1* and *MYC* in 11 group 3 medulloblastoma (MB) patient samples from a microarray dataset of University of Colorado. (**b**) Correlation of mRNA levels between *PLK1* and *MYC* in 134 group 3 MB patient samples of the Cavalli dataset [5]. (**c**) The real-time PCR analysis of *c-MYC* expression upon *c-MYC* shRNA knockdown for 72 h in the D425 and D458 cell lines; Mean  ±  SD; *** *p*  <  0.001 (one-way ANOVA). (**d**) Real-time PCR analysis of *PLK1* expression upon *c-MYC* shRNA knockdown for 72 h in the D425 and D458 cell lines; Mean  ±  SD; *** *p*  <  0.001 (one-way ANOVA). (**e**) The immunoblot detection of c-MYC and PLK1 protein levels after 72 h of *c-MYC* shRNA transfection, with β-actin as a loading control. The quantification plot can be found in Figure S2a. (**f**) Induction of omoMYC in D458 cells were determined by Western blotting. Levels of PLK1 was determined by Western blot. β-actin was used as a loading control. The quantification plot can be found in Figure S2b. (**g**) E-box motif on promoter regions of *PLK1* (top). IGV screenshot of representative chromatin immunoprecipitation (ChIP)-Seq data of c-MYC on the promotion of *PLK1*. Data shown for the D458 cell lines from published studies (bottom). Genome-wide analyses of c-MYC occupancy demonstrated that c-MYC binds to the promoter region of *PLK1* gene. A IgG isotype control was used for the control. K562: leukemia line; A549: lung carcinoma cell line; hESC: human embryonic stem cell lines; and MCF-7: breast cancer cell line. (**h**) Binding of c-MYC to the promoter of *PLK1* analyzed by ChIP PCR in D458 cells. *CDK4*-peak was used for the positive control. Mean  ±  SD; *** *p*  <  0.001 (one-way ANOVA). The original blots are in Figure S3.

We previously demonstrated that *PLK1* is highly expressed in MYC-driven medulloblastoma and that the inhibition of PLK1 with BI2536 suppresses tumor cell growth [12]. To evaluate the mechanisms by which *PLK1* is overexpressed in MYC-driven medulloblastoma, we first asked whether c-MYC activates *PLK1* transcription in medulloblastoma. We examined the expression of *PLK1* and *c-MYC* in two cohorts of patient samples [5,22]. Microarray results showed that the expression of *c-MYC* and *PLK1* was positively correlated in medulloblastoma (Figure 1a,b). We then depleted *c-MYC* with two specific shRNAs in the MB cell lines D425 and D458 (Figure 1c and Figure S1). *C-MYC* knock-down caused a significant reduction in *PLK1* mRNA and protein levels in both D425 or D458 cells (Figure 1d,e). These data suggested that MYC directly induces *PLK1* transcription in medulloblastoma.

To confirm that MYC activates the transcription of *PLK1*, we next transfected D458 cells with omoMYC, which is a dominant-negative MYC inhibitor that inhibits the transcriptional activation of MYC target genes by preventing MYC heterodimerization with MAX [23]. Doxycycline was administered to induce the expression of omoMYC. Notably, the inhibition of the MYC protein led to marked decreases in the level of the PLK1 protein (Figure 1f). Additionally, we identified a MYC E-box binding motif at 198 base pairs upstream of the PLK1 transcriptional start site. Chromatin immunoprecipitation (ChIP)-sequencing in D458 cells revealed a significant increase in MYC recruitment to the *PLK1* promoter-proximal E-box motif compared with a IgG isotype control (Figure 1g). MYC binding to the *PLK1* promoter was further confirmed using public data from Encyclopedia of DNA Elements (ENCODE) (Figure 1g). The chromatin occupancy profiles were also verified in a ChIP PCR assay (Figure 1h).

### 2.2. PLK1 Antagonizes FBXW7-Mediated Degradation of c-MYC

**Figure 2:**
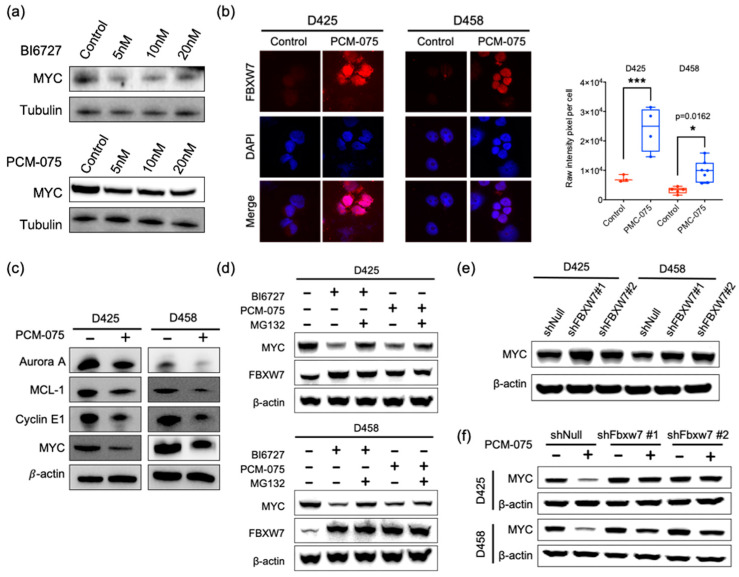
PLK1 antagonizes FBXW7-mediated degradation of c-MYC. (**a**) Western blot of c-MYC and tubulin with various concentrations of 5–20 nM BI6727 or PCM-075 treatment in D458 cells for 48 h. The quantification plot can be found in Figure S2c. (**b**) Representative immunofluorescence of FBXW7 in D425 and D458 cells treated with 10 nM PCM-075 for 48 h. Results are representative of three independent experiments. (**c**) Western blot of AURORA A, MCL-1, Cyclin E1, c-MYC and β-actin. D425 and D458 cells were treated with 10 nM PCM-075 for 48 h. The protein levels were analyzed by immunoblot, with β-actin as a loading control. (**d**) PLK1 sustains MYC through FBXW7. D425 and D458 cells were treated with BI6727 (10 nM) or PCM-075 (10 nM) for 48 h. For MG132 treatment, cells were treated with MG132 (10 mM) for 6 h before harvest. FBXW7 and c-MYC protein levels were analyzed by immunoblot with β-actin as a loading control. (**e**,**f**) FBXW7 depletion rescued the c-MYC loss resulting from PLK1 inhibition. D425 or D458 cells were infected with the control shRNA or validated shRNAs targeting FBXW7 for 72 h and then treated with or without PCM-075 (10 nM) for 48 hr. The c-MYC levels were analyzed by immunoblot, with β-actin as a loading control. The original blots are in Figures S3 and S4. * *p*  <  0.05, *** *p* <  0.001.

A PLK1/FBXW7/N-MYC pathway has been demonstrated in neuroblastoma, where PLK1 phosphorylates FBXW7, promotes FBXW7 degradation and leads to the stabilization of N-MYC [24]. In order to test whether a similar mechanism exists in c-MYC-driven medulloblastoma, we examined c-MYC protein levels in MB D458 cells treated with PLK1 inhibitors PCM-075 and BI6727. PLK1 inhibitor treatment led to the loss of c-MYC protein levels, as determined by Western blot (Figure 2a). Immunofluorescence showed FBXW7 protein abundance was significantly increased in the PCM-075 treated D425 and D458 cell lines (Figure 2b). Additional targets of FBXW7, including AURORA A MCL-1, and Cyclin E, were also downregulated in response to PCM-075 in MB cells, implying FBXW7 is an upstream regulator of c-MYC in medulloblastoma (Figure 2c). Moreover, the administration of BI6727 or PCM-075 in D425 and D458 cells consistently increased endogenous FBXW7 levels concomitant with the degradation of c-MYC. The degradation was rescued by the addition of the 26S proteasome inhibitor MG132, suggesting a posttranslational regulation of c-MYC via PLK1 (Figure 2d). The knockdown of FBXW7 in the D425 and D458 cell lines increased c-MYC and abolished the PCM-075-induced loss of the c-MYC protein, supporting our conclusion that FBXW7 mediates the degradation of c-MYC, while PLK1 promotes c-MYC stabilization via the abrogation of the FBXW7 in MYC-amplified medulloblastoma cell lines (Figure 2e,f).

### 2.3. PLK1-MYC-FBXW7 Regulatory Loop in Medulloblastoma

**Figure 3:**
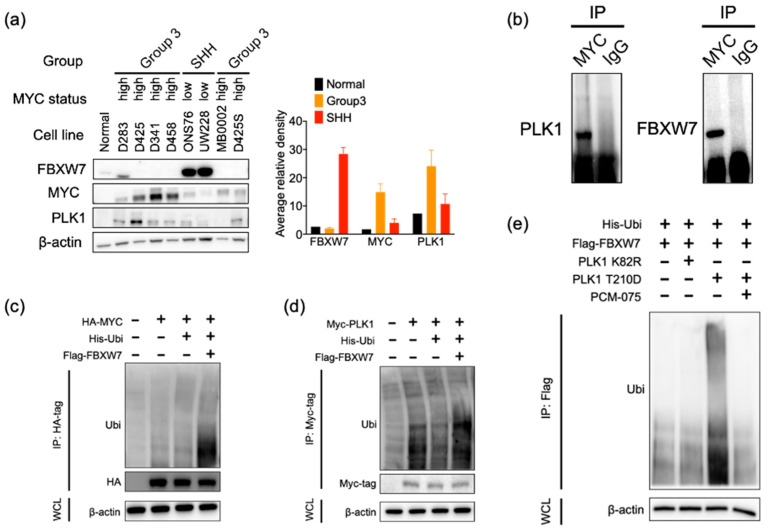
PLK1-MYC-FBXW7 regulatory loop in medulloblastoma. (**a**) Western blot analysis of FBXW7, c-MYC, and PLK1 in medulloblastoma cell lines. The protein was normalized with β-actin on the same membrane (left). Quantification bar blot of Western blots (right). The quantification for each protein can be found in Figure S2. (**b**) Endogenous interaction between c-MYC with PLK1 and FBXW7. Lysates from D458 cells were subjected to immunoprecipitation using an anti-c-MYC antibody, and proteins that co-precipitated with c-MYC were detected by immunoblot using anti-PLK1 or anti-FBXW7 antibodies. (**c**,**d**) FBXW7 promotes PLK1 and c-MYC ubiquitination. The HA293 cells were transfected with plasmids expressing Myc tag PLK1 or HA tag c-Myc, His-ubiquitin and Flag tag FBXW7. Cell lysates were immunoprecipitated with specific Myc tag or HA tag antibodies, and the ubiquitin protein levels were analyzed by immunoblot. β-actin was used as a loading control. (**e**) FBXW7 poly-ubiquitination in the presence or absence of PLK1 mutants. HEK293 cells were transfected with plasmids expressing ubiquitin, Flag tag FBXW7 and constitutively active PLK1 (PLK1-T210D) or kinase inactive mutant PLK1 (PLK1-K82R), as indicated, followed by lysis in an IP buffer. A ubiquitin-conjugated FBXW7 protein was immunoprecipitated with a FLAG tag antibody and subjected to an immunoblot assay with a ubiquitin antibody. β-actin was used as a loading control. The original blots are in Figure S5.

We then evaluated the expression of MYC, PLK1, and FBXW7 in a panel of well-characterized medulloblastoma cell lines (Figure 3a). Group 3 cell lines expressed lower levels of the FBXW7 protein and higher levels of the PLK1 and MYC proteins compared with the SHH group or normal cerebellum, further confirming the inverse relationship of FBXW7 and MYC/PLK1 in medulloblastoma.

To study the interaction of PLK1-FBXW7 and MYC-FBXW7, we performed immunoprecipitation and electrophoresis with the c-MYC antibody in D458 cells. Western blot analysis demonstrated the endogenous interaction of MYC-FBXW7 and MYC-PLK1 (Figure 3b). When we co-expressed flag-FBXW7 with HA-MYC or myc-tag-PLK1 in HEK293 cells, immunoprecipitation with an antibody myc-tag or an HA-tag antibody showed that FBXW7 promoted the ubiquitination of PLK1 and c-MYC. Together, these data indicated that FBXW7 physically interacts with PLK1 and c-MYC, and it induces the ubiquitination and proteasome degradation of PLK1 and c-MYC in medulloblastoma (Figure 3c,d).

FBXW7 has been found to be regulated by proteasomal degradation through self-poly-ubiquitination [24]. Our results demonstrated that PLK1 inhibition enhanced FBXW7 stability in medulloblastoma. In order to determine whether PLK1 promotes FBXW7 degradation through phosphorylation, we transfected constitutively active PLK1 (PLK1-T210D) and kinase inactive mutant PLK1 (PLK1-K82R) in HEK293 cells. PLK1-T210D, but not PLK1-K82R, increased the ubiquitination level of FBXW7. Treatment with PCM-075 decreased FBXW7 poly-ubiquitination by inhibiting PLK1 activity, suggesting that PLK1 activation is required for destabilizing FBXW7 and that PLK1 inhibition stabilizes c-MYC by regulating FBXW7 auto-ubiquitination (Figure 3e).

### 2.4. FBXW7 Is Descreased in Medulloblastoma

**Figure 4:**
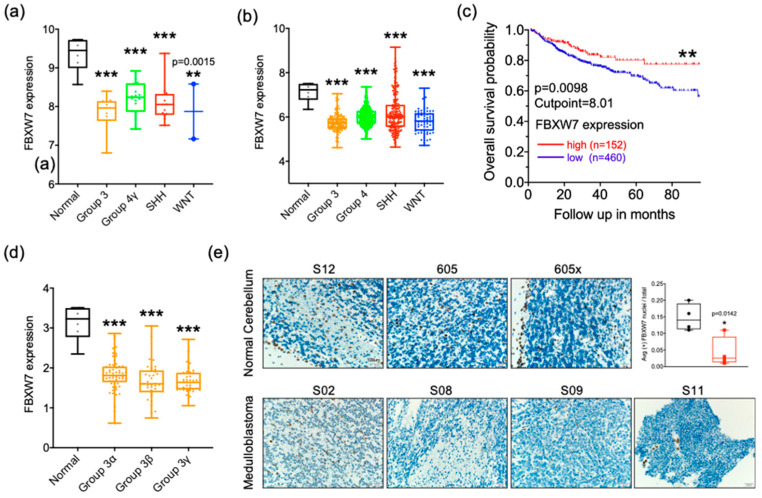
FBXW7 is decreased in medulloblastoma. (**a**) Microarray analysis showing *FBXW7* expression in 44 medulloblastoma patient samples. *n* = 6 in normal, *n* = 11 in group 3, *n* = 18 in group 4, *n* = 13 in the SHH group, and *n* = 2 in the WNT group. Mean  ±  SD; ** *p* < 0.01; *** *p*  <  0.001 (one-way ANOVA). (**b**) Microarray analysis showing *FBXW7* expression in 763 medulloblastoma patient samples of Cavalli data. *n* = 6 in normal, *n* = 134 in group 3, *n* = 317 in group 4, *n* = 211 in the SHH group, and *n* = 65 in the WNT group. Mean  ±  SD; ** *p* < 0.01; *** *p*  <  0.001 (one-way ANOVA). (**c**) Kaplan–Meier plots indicating overall survival in relation to *FBXW7* expression in all MB patient populations. ** *p* < 0.01, Log-rank (Mantel–Cox) test. (**d**) Microarray analysis showing *FBXW7* expression in 134 group 3 medulloblastoma patient samples of Cavalli data. *n* = 61 in group 3α, *n* = 35 in group 3β, and *n* = 38 in group 3γ. Mean  ±  SD; ** *p* < 0.01; *** *p*  <  0.001 (one-way ANOVA). (**e**) IHC staining of human group 3 medulloblastoma and normal cerebella tissue using a specific antibody for FBXW7. Scale bar: 1 mm. Images shown at 40×. Mean  ±  SD; two-tailed student *t* test, * *p* < 0.05.

FBXW7 is responsible for degrading diverse oncoproteins and is considered a tumor suppressor in many types of cancers. To establish the role of FBXW7 in medulloblastoma, we performed microarray analysis in 44 MB patient samples and six normal cerebella samples (Figure 4a). We also examined the expression of *FBXW7* in a cohort of 763 recently described MB samples (Figure 4b). Microarray data generated from two platforms were normalized and merged in order to generate a combined series that would facilitate the analyses. The results showed that *FBXW7* is significantly downregulated in all subgroups of MB. Kaplan–Meier survival curves showed that high *FBXW7* expression significantly correlates with a better overall survival (Figure 4c). Moreover, all subtypes of group 3 MB samples were found to express notably lower *FBXW7* levels than normal cerebellum, including in the high *c-MYC* expressed group 3 subtype (Figure 4d). We then examined group 3 pediatric patient samples by immunohistochemistry staining (Figure 4e). FBXW7 was considerably decreased in tumor tissues compared with the normal human cerebellum. These results suggested that FBXW7 is an important mediator in the tumorigenesis of medulloblastoma.

### 2.5. FBXW7 Overexpression Increases Apoptosis in Medulloblastoma Cells

**Figure 5:**
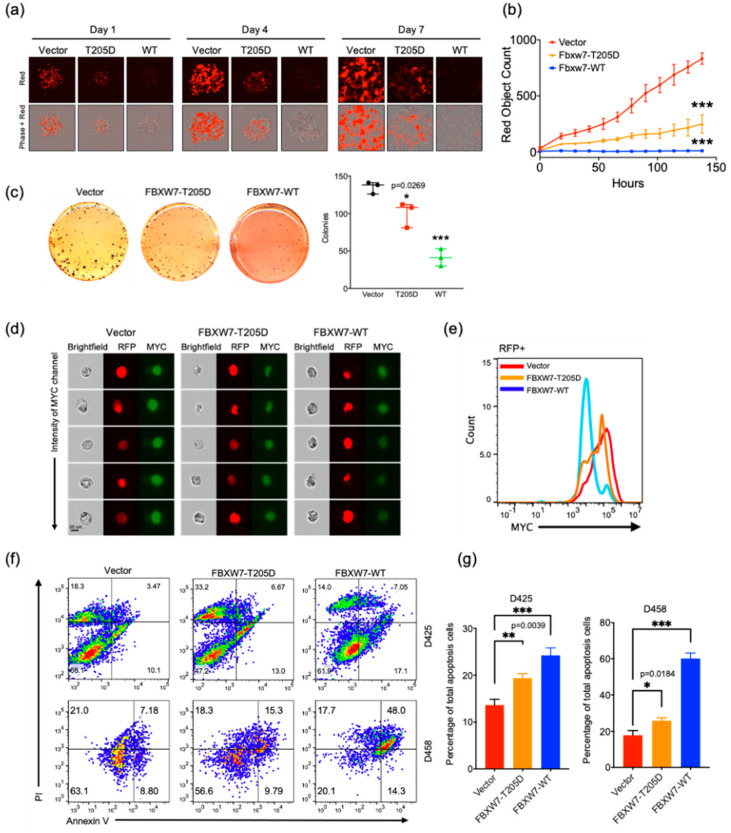
FBXW7 overexpression increases apoptosis in medulloblastoma cells. (**a**) Representative immunofluorescence images of RFP in D458 cells expressing *FBXW7* (wild-type or T205D mutant) or a pLenti-CMV-RFP-2A-Puro-Blank vector. Results are representative of five independent experiments on an Incucyte system for live cell imaging with 2000 ng/mL of puromycin. (**b**) Cell growth assay in D458 cells expressing *FBXW7* (wild-type or T205D mutant) or a vector (*n* = 3). Mean  ±  SE; *** *p*  <  0.001 (one-way ANOVA). (**c**) Methylcellulose assay of D458 cells expressing *FBXW7* (wild-type or T205D mutant) or a vector. The experiment was performed in triplicate. Mean  ±  SD; * *p*  <  0.05; *** *p*  <  0.001 (one-way ANOVA). (**d**) Images ordered by the top intensity of the MYC signal acquired from Amnis FlowSight. The D458 cells expressing *FBXW7* (wild-type or T205D mutant) or a vector (*n* = 3) were stained with an MYC antibody and gated by an RFP-positive signal. (**e**) Amnis FlowSight analysis of c-MYC expression in RFP-positive cells of the D458 cell line expressing *FBXW7* (wild-type or T205D mutant) or a vector. (**f**,**g**) Flow cytometry analysis of early and late apoptosis in the D425 or D458 cell lines expressing *FBXW7* (wild-type or T205D mutant) or a vector. The cells were analyzed after staining with FITC-conjugated annexin V and PI by a flow cytometer. Quantification of apoptotic cell percentage in the D425 or D458 cell lines expressing *FBXW7* (wild-type or T205D mutant) or a vector (*n* = 3). Mean  ±  SD; * *p*  <  0.05; ** *p*  <  0.01; *** *p*  <  0.001 (one-way ANOVA).

Recent studies have demonstrated that kinases phosphorylate FBXW7 at Thr205 and promote its proteasomal degradation [25,26]. In order to evaluate whether Thr205 residue phosphorylation affects the function of FBXW7 in medulloblastoma, we constructed WT-FBXW7 and phosphomimetic aspartic acid mutant (T205D) and performed a cell proliferation assay in D458 cells. In comparison with the vector control, wild-type *FBXW7* dramatically decreased the proliferation of MB cells. The phosphomimetic T205D mutation decreased proliferation but to a lesser extent than the WT-FBXW7, suggesting that phosphorylation fosters the ubiquitination and subsequent degradation of FBXW7 (Figure 5a,b). To determine if overexpressed *FBXW7* impairs the ability of MB cells to form adhesion-independent colonies, we performed a methylcellulose assay. WT-FBXW7 or *FBXW7* with T205 mutation-transduced D458 cells were plated in 1.3% methylcellulose, and colonies were counted after 14 days. WT-FBXW7 overexpressing cells showed a more than 50% reduction in the number of colonies compared with the vector-transduced cells, whereas T205D mutation weakened this trend (Figure 5c). As expected, the cells transduced with WT-FBXW7 showed a notably lower MYC signal compared with the cells transduced with a vector or the T205 mutation, supporting the notion that FBXW7 mediates MYC degradation in MB cells (Figure 5d,e).

To determine whether the detected reduction in proliferation was due to a cessation of growth or an increase in cell death, we examined annexin V positivity by flow cytometry. D425 and D458 cells overexpressed with WT-FBXW7 showed a significant increase in annexin V (+), and the T205D mutation also demonstrated an increase in annexin V (+) but to a lesser extent than WT-FBXW7, indicating that overexpressed *FBXW7* improved the sensitivity of MB cells to apoptosis (Figure 5f,g).

### 2.6. Activation of FBXW7 Is a Potential Therapeutic Strategy for c-MYC-Driven Medulloblastoma

**Figure 6:**
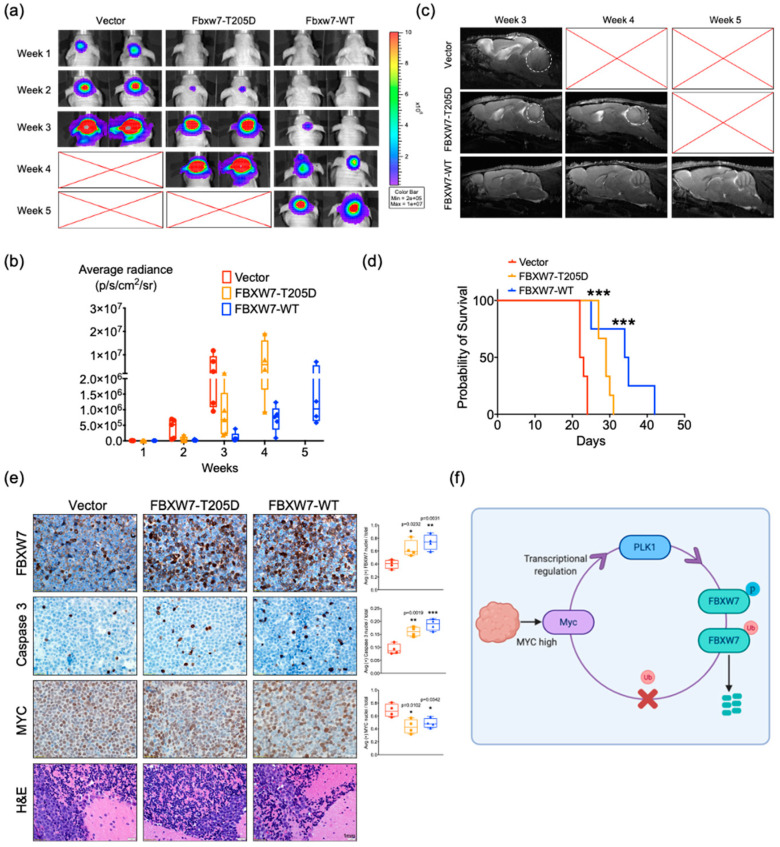
Activation of FBXW7 is a potential therapeutic strategy for MYC-driven medulloblastoma. (**a**,**b**) Representative images of bioluminescence imaging from nude mice xenografts injected with D458 cells expressing *FBXW7* (wild-type or T205D mutant) or a vector. *n* = 7 for each group. (**c**) Representative sagittal T2-weighted turboRARE (rapid acquisition with relaxation enhancement) MR images on cerebellar D458 tumor lesions expressing *FBXW7* (wild-type and T205D mutant) or a vector. (**d**) Kaplan–Meier survival plot from intracranial orthotopic mouse model. *** *p* < 0.001, Log-rank (Mantel–Cox) test. (**e**) Immunohistochemical staining of MB from intracranial orthotopic mouse model for FBXW7, c-MYC, Caspase 3, and H&E. Images taken at 40×. Mean  ±  SD; * *p*  <  0.05; ** *p*  <  0.01; *** *p*  <  0.001 (one-way ANOVA). (**f**) Proposed model depicting the PLK1-MYC-FBXW7 signaling circuits. PLK1 promotes FBXW7 auto poly-ubiquitination and proteasomal degradation, counteracting the FBXW7-mediated degradation of c-MYC. In turn, stabilized c-MYC directly activates PLK1 transcription, constituting regulatory loops.

To examine the effect of FBXW7 in vivo, luciferase-expressing D458 cells with *FBXW7* constructs (empty vector, WT-FBXW7, or FBXW7-T205D mutation) were injected into the cerebellum of mice, and tumor growth was monitored in vivo. Animals in the vector group showed a rapid increase of the bioluminescence signal in week 1, the T205D mutation group showed the bioluminescence signal after week 2, and the WT-FBXW7 group showed the signal after week 3 (Figure 6a,b). We also assessed tumor volumes by high-resolution T2-weighted MRI. We found that the tumor volume was larger in the vector group, while both the mutation and WT-FBXW7 groups exhibited a slowed growth of tumors (Figure 6c). Consistent with bioluminescence imaging and MRI results, mice bearing WT-FBXW7 and FBXW7-T205D revealed an enhanced survival compared to mice bearing the same cells with a vector (Figure 6d). These results demonstrated that FBXW7 is critical for blocking tumor progression, and T205 phosphorylation promotes its degradation and abolishes FBXW7 tumor suppressor function in medulloblastoma.

Additionally, an immunohistochemical analysis displayed an overexpression of FBXW7 and depleted c-MYC expression concomitant with massive intertumoral apoptosis as quantified by c-caspase-3 staining (Figure 6e). Taken together, all of these results suggest that the overexpression of FBXW7 confers a growth disadvantage in MB cells and the activation of FBXW7 is a potential therapeutic strategy against c-MYC-driven MB.

## 3. Discussion

FBXW7 is considered to be a strong tumor suppressor that governs human cell cycle progression, cell growth, and tumor development by directing certain oncoproteins to ubiquitin-mediated proteolysis. It is frequently deactivated by mutations or genetic deletions in many types of cancer [19,27]. However, a previous study reported that *FBXW7* mutations are not frequently observed in group 3 MB, implying that wild-type FBXW7 is deregulated by a different mechanism [28]. In this study, we proved that PLK1 directly interacts with FBXW7, fostering its phosphorylation and auto-polyubiquitination in MB. We also demonstrated the tumor suppressor role of FBXW7 in group 3 MB. By overexpressing wild-type and mutant forms of FBXW7, we observed a remarkable growth disadvantage in MB, both in vitro and in vivo. Our study identified FBXW7 as a critical suppressor in the tumorigenesis of medulloblastoma.

Phosphorylation residues of FBXW7, as well as the surrounding amino acids, are highly conserved among vertebrates, indicating that the phosphorylation of these sites may have an evolutionarily conserved role in the regulation of FBXW7 stability [19,26]. Thr205 is an important and most well-known phosphorylation site of FBXW7. The extracellular signal-regulated kinase (ERK) can interact with and phosphorylate FBXW7 at Thr205, leading to FBXW7 ubiquitination and proteasome-mediated degradation [25]. Pin1 also negatively regulates FBXW7 stability through T205 [26]. In our study, both in vivo and in vitro data showed that phosphorylation at the T205 site inhibited FBXW7 tumor suppressor function in MB. Furthermore, a previous study showed that FBXW7 governs cellular apoptosis by targeting the pro-survival Bcl-2 family member, Mcl-1, for ubiquitination and destruction in a GSK3 phosphorylation-dependent manner [29]. Here, we revealed that PLK1 inhibition increased FBXW7 protein abundance accompanied by a decrease of MCL-1. We also showed *FBXW7* overexpression increased apoptosis. Thus, FBXW7 may modulate apoptosis by promoting MCL-1 degradation in medulloblastoma.

Direct pharmacological approaches to the inhibition of MYC family members has been proven difficult. Our findings showed that targeting PLK1 signaling provokes MYC destruction by the proteasome, inducing a robust apoptotic therapeutic response. Moreover, FBXW7 has also been reported to ubiquitylate and degrade through the phosphorylation by GSK3β [30], while AURORA A is targeted for ubiquitination and subsequent degradation by FBXW7 in a process that is regulated by GSK3β [31,32]. In addition, ERK phosphorylates and destabilizes FBXW7 in pancreatic cancer [25]. Thus, many kinases can negatively regulate FBXW7 stability by promoting its self-ubiquitination, which indicates a potential therapeutic strategy against MYC-driven cancer.

Due to the role of PLK1 in the cell-cycle and kinase pathway that are significant to cancer progression, PLK1 is recognized as a ‘druggable target’ for the development of therapeutics for the management of a variety of cancers [33,34,35]. There are several clinical trials going on now [36]. Our results strongly supported the idea that PLK1 holds promise as a therapeutic target in MB and revealed that PLK1 inhibition can reduce tumor cell proliferation and increase apoptosis in MB. The inhibition of PLK1 with PCM-075 or BI6727 decrease the phosphorylation of FBXW7, reducing its poly auto-ubiquitylation and resulting in an accumulation of FBXW7. The accumulation of FBXW7 facilitates the E3 ubiquitin ligases degradation of MYC and the MCL-1 protein. Moreover, we demonstrated that MYC also actives *PLK1* transcription (Figure 6f). Collectively, these results suggest that a FBXW7-MYC-PLK1 signaling circuit underlies the tumorigenesis of MB and validate PLK1 inhibitors as potentially effective therapeutics for MYC-overexpressing cancers.

## 4. Materials and Methods

### 4.1. Cell Lines and Reagents

The D341, D425, and D458 cell lines were provided by Darell D. Bigner (Duke University Medical Center, Durham, NC, USA). The small molecule PLK1 inhibitor BI6727 was purchased from Chemitek (Indianapolis, IN, USA), and PCM-075 was provided by Trovagene (San Diego, CA, USA). The drugs were reconstituted in dimethyl sulfoxide (DMSO). An equivalent amount of DMSO for the highest concentration of drug was used for each experiment as a vehicle control.

### 4.2. Plasmids

The *FBXW7* lentiviral vector (#LV158451) and the pLenti-CMV-RFP-2A-Puro-Blank vector (#LV591) were purchased from ABMgood (Richmond, BC, Canada). The T205D mutation was cloned into the lentiviral vector to generate *FBXW7* expression plasmids. The HEK293T cell line was used to produce lentivirus-expressing vectors. Briefly, the transfection was performed by using the Lipofectamine 3000 Transfection Reagent (Invitrogen, Carlsbad, CA, USA). After 18 h post transfection, the media were removed and replaced with fresh media. The lentivirus was harvested the next day and used to generate stable cell lines (D425 and D458). The transduced cells were selected with 2000 ng/mL of puromycin for 48 h. The same concentration of puromycin was added to the growth medium during the whole experiment.

### 4.3. Quantitative Real-Time Polymerase Chain Reaction

RNA was isolated using a Qiagen RNeasy kit (Valencia, CA, USA). cDNA was synthesized from 2 μg of total RNA with High-Capacity cDNA Reverse Transcription Kit (Thermo Fisher Scientific). Real-time PCR was performed using Power SYBR-Green PCR mastermix (Thermo Fisher Scientific). qPCR was performed on a StepOnePlus Real-Time PCR system (Thermo Fisher Scientific). The primer sequences were as follows: CDK4-peak, ATGGCTACCTCTCGATATGAGC and CATTGGGGACTCTCACACTCT; PLK1-peak, GCCCGAGAAAGGGAGAAAC and ATAGCCTGGGAAACCAAACC; and PLK1, CACCAGCACGTCGTAGGATTC and CCGTAGGTAGTATCGGGCCTC.

### 4.4. Microarray Preparation and Data Processing

RNA from all surgical specimens was extracted, amplified, labeled, and hybridized to Affymetrix HG-U113 plus 2 microarray chips (Affymetrix, Santa Clara, CA, USA). The scanned microarray data were background-corrected and normalized using the RMA algorithm, resulting in log 2 gene expression values. For public microarray data, raw CEL files were downloaded from the Gene Expression Omnibus under accession numbers GSE85217 and normalized using the RMA algorithm. The gene expression array data generated using the Affymetrix Gene 1.1 ST array (Santa Clara, CA, USA) and U133 Plus 2.0 array platforms were merged in order to generate a combined value. For each platform, a contrast value per gene was calculated by subtracting the mean expression of that gene across all samples hybridized on that platform from each individual, and the resulting contrast values of the two platforms were then combined.

### 4.5. ChIP-Sequencing

ChIP-seq libraries were sequenced on the Illumina Novaseq 6000 platform. Bowtie2 was used to align the 150-bp paired-end sequencing reads to a reference human genome (hg38) downloaded from the UCSC Genome Browser. Peaks were called using MACS2 (v2.1.1.20160309) with default parameters [37]. Peak locations were further annotated according to the known genes in hg38, and 3000 bp of upstream and downstream of transcription start sites were considered as promoter regions using the R/Bioconductor package ChIPseeker [38].

### 4.6. Western Blotting and Immunoprecipitation

Cells were lysed in a RIPA buffer (Thermo Fisher Scientific, Waltham, MA, USA) containing an EDTA-free protease inhibitor (Roche Diagnostics, Basel, Switzerland), and protein concentrations were determined with the BCA Protein Assay Kit (Pierce, Thermo Fisher Scientific). Protein (30 μg in total) was separated on a 4–20% SDS-PAGE gradient (Bio-Rad). The membrane was incubated with a primary antibody overnight at 4 °C. A secondary antibody—α-mouse-HRP (#7076, cell signaling), α-rabbit-HRP (#7074, cell signaling), or α-actin-HRP (#12262, cell signaling)—was exposed for 1 h at room temperature. Blots were developed with Luminata Forte Western HRP (Millipore) and imaged using Syngene GBox Chemi-SL1.4 gel doc. Antibodies used for Western blot analysis were from the following sources: β-actin #8457, Aurora A #14475, Mcl-1 #94296, and Cyclin E1 #4219 were purchased from Cell Signaling, USA, and PLK1 ab17056, c-MYC ab32072, c-MYC ab39688, c-MYC ab185655 (phopho S58) ab185656, c-MYC (phopho S62) ab185656, FBXW7 ab109617, and ubiquitin ab7780 were purchased from Abcam. Western blots were quantified using ImageJ.

Immunoprecipitation assays were performed by using an anti-c-MYC antibody, a PLK1 antibody, an HA-tag antibody (Y1070, UBPBio, Aurora, CO, USA), a Myc-tag antibody (Y1090, UBPBio), a DYKDDDDK-tag antibody (Y1101, UBPBio), and Dynabeads (Thermo Fisher, Waltham, MA, USA). Immunoprecipitations with IgG were used as controls for specificity.

### 4.7. Immunofluorescence

Three thousand D425 or D548 cells grown in poly-d-lysine-coated chamber slides were treated with an 10nM PCM-075 or DMSO for 48 h. After treatment, cells were washed and fixed with 4% paraformaldehyde for 15 min at room temperature. Cells were then permeabilized with 0.2% Triton X-100 in PBS for 15 min followed by incubation in 5% milk diluted in 0.05% Triton X-100 for 30 min at room temperature on a shaker. After blocking, cells were incubated with the primary antibodies. The FBXW7 antibody was used at a dilution of 1:200 for 1 h at room temperature. After washing with 0.05% Triton X-100 (3 times for 5 min each), cells were incubated with an Alexa Fluor 647-conjugated secondary antibody (1:500) for 1 h at room temperature in the dark, washed with PBS (3 times for 5 min each), and mounted using a ProLong Gold antifade reagent containing DAPI (Sigma, St. Louis, MO, USA). Images were acquired using an inverted epifluorescence microscope at a magnification of 20×.

### 4.8. Immunohistochemistry

For histology, tumors from patient samples or experimental mice were dissected and either frozen or preserved in 10% formalin. The samples were rinsed in PBS and fixed in 4% paraformaldehyde overnight at 4 °C and embedded in paraffin. Antigen retrieval was performed by the application of a citrate buffer with a pH of 6.00 for 20 min. Slides were then incubated with FBXW7, c-MYC, PLK1, cleaved caspase 3 antibodies, and H&E overnight at 4 °C. The secondary antibody conjugated to horseradish peroxidase was applied and detected using the Dako Envision Kit for 3,3′-diaminobenzidine. All patients provided written informed consent for molecular studies of their tumor, and the protocol was approved by the ethics committee of University of Colorado and Children’s Hospital Colorado (COMIRBs #95–500).

### 4.9. Flow Cytometry Assay

Cells were seeded in 10 cm plates (106 cells/well) and treated with 10 nM of BI6727 or PCM-075. Cells were harvested 48 h later and fixed with 4% formaldehyde for 15 min at room temperature. Fixed cells were then washed and permeabilized with methanol on ice for 10 min. The cells were stained with a c-MYC (#5605, cell signaling) antibody. The flow cytometric analysis was performed on the Amnis FlowSight flow cytometer (Millipore, Burlington, MA, USA).

### 4.10. Cell Apoptosis Assay

Cells were transfected with a vector, T205D-FBXW7, and WT-FBXW7. Equal numbers of cells were then stained using a Guava Nexin reagent (Millipore) to detect apoptotic cells.

### 4.11. Methylcellulose Assays

In a 1:1 mixture of 2.6% methylcellulose and complete growth medium, 500 cells/3 mL were plated. Cells were allowed to grow for ten days. Colonies were stained with nitrotetrazolium blue chloride (Sigma) at 1.5 mg/mL in PBS for 24 hrs at 37 °C and then counted.

### 4.12. In Vivo Xenograft Experiments

D458 cells were collected and resuspended as a single cell suspension of 20,000 cells/3 μL in serum free media. Cell injection and the following animal experiment was performed as previously described [39]. Tumor bioluminescence was analyzed using the Living Image 2.60.1 software (Caliper Life Sciences, PerkinElmer, Waltham, MA, USA). Animal care and experimental procedures were conducted in accordance with the guidelines of the University of Colorado Center for Comparative Medicine and the University of Colorado Institutional Animal Care and Use Committee (protocol number: 00052).

### 4.13. Magnetic Resonance Imaging

For in vivo MRI acquisitions, mice were anesthetized shortly before and during the MR session using a 1.5% isoflurane/oxygen mixture. Anesthetized mice were placed on a temperature-controlled mouse bed below a mouse head array coil and inserted into a Bruker 9.4 Tesla BioSpec MR scanner (Bruker Medical, Billerica, MA, USA). First, T2-weighted turboRARE images were acquired using the following parameters: repetition time (TR) = 3268 ms; echo time (TE) = 60 ms; RARE factor = 12 and 8 averages; FOV = 20 mm; matrix size = 350 × 350; slice thickness = 700 μm; 24 sagittal and axial slices; and in-plane spatial resolution = 51 μm. Then, a diffusion-weighted EPI sequence with 6 b values was used using 4 axial slices covering all tumor lesions and unaffected brain tissue. Tumor regions were manually segmented on T2-weighted images by placing hand-drawn regions of interest (ROI), and the volume was calculated as mm^3^. The apparent diffusion coefficients (ADC; s/mm^2^) were calculated from diffusion-weighted imaging maps as a criterion for tumor cellularity. All acquisitions and image analysis were performed using the Bruker ParaVision NEO software (Bruker, Billerica, MA, USA).

### 4.14. Statistics

Statistical analysis was performed using the GraphPad Prism 8 software (GraphPad, San Diego, CA, USA). One-way ANOVA tests and two-tailed Student’s *t*-tests were used for comparisons between groups. A log-rank (Mantel–Cox) test was use for survival curve comparison. *p*-values < 0.05 were considered to indicate significance.

## 5. Conclusions

Our study found FBXW7 is decreased in group 3 MB, MYC directly activates the transcription of *PLK1*, and PLK1 inhibition leads to the degradation of MYC by stabilizing FBXW7. These results demonstrated the FBXW7-MYC-PLK1 regulatory loop and that FBXW7 stabilization is crucial for the suppression of tumorigenesis in MYC-driven medulloblastoma.

## Data Availability

Publicly available datasets were analyzed in this study. The data can be found on Gene Expression Omnibus: GSE85217 and GSE68015.

## Supplementary Materials

The following are available online at https://www.mdpi.com/2072-6694/13/3/387/s1, Figure S1: Proliferation of D425 and D458 shNull or shMYC cell lines, Figure S2: Western blot analysis and quantification, Figure S3: Full blots corresponding to Figure 1 and Figure 2e, Figure S4: Full blots corresponding to Figure 2, Figure S5: Full blots corresponding to Figure 3.
