# Supplementary material for: A Regulatory Loop of FBXW7-MYC-PLK1 Controls Tumorigenesis of MYC-Driven Medulloblastoma
Source: Cancers (Basel). 2021 Jan 21;13(3):387. doi: 10.3390/cancers13030387 (PMC7865656; doi:10.3390/cancers13030387)
Supplement: Supplementary file 1 [file cancers-13-00387-s001.pdf]

Supplementary Materials: A Regulatory Loop of FBXW7-MYC-PLK1 Controls Tumorigenesis of MYC-Driven Medulloblastoma

Dong Wang, Angela Pierce, Bethany Veo, Susan Fosmire, Etienne Danis, Andrew Donson, Sujatha Venkataraman and Rajeev Vibhakar

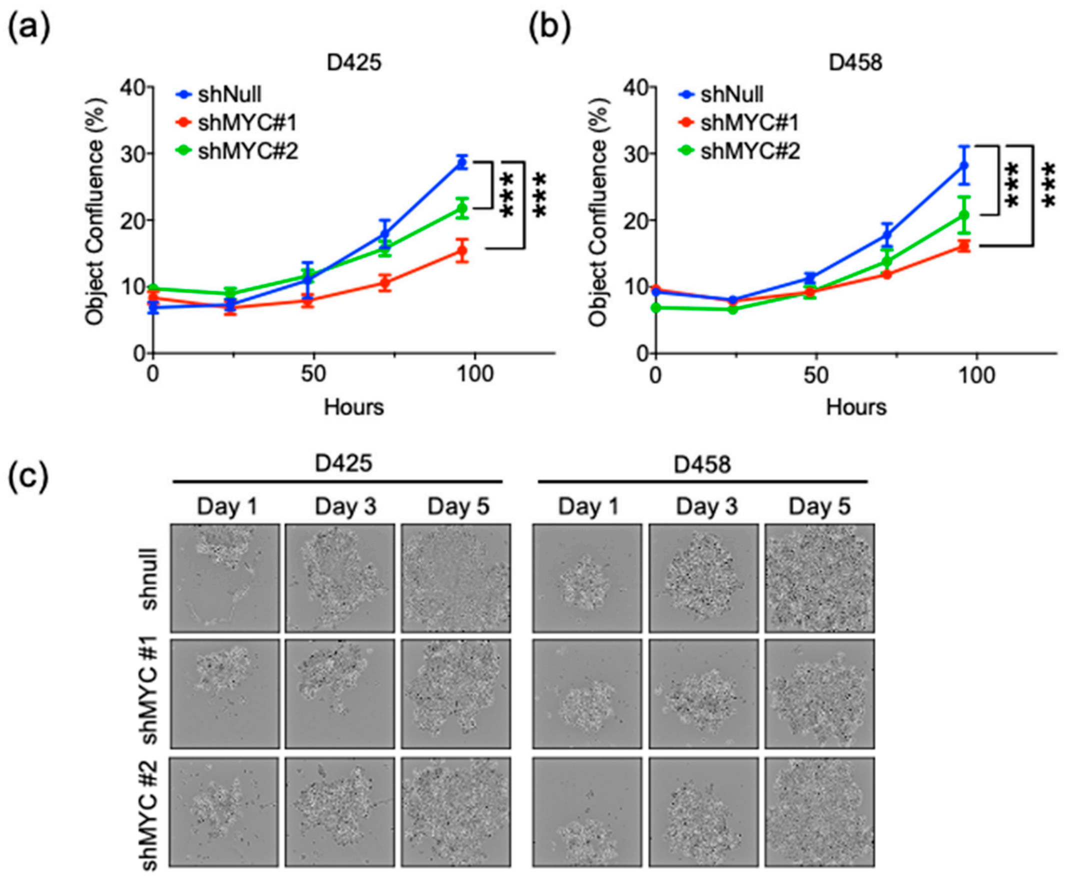

Figure S1. Proliferation of D425 and D458 shNull or shMYC cell lines. (**a-b**) Proliferation of D425 and D458 shNull or shMYC cell lines (n = 5). The cells grew in Incucyte system for live cell imaging with 2000ng/ml puromycin. (**c**) Representative images of cell growth assay in D425 or D458 cell lines expressing shNull or shMYC. *** $p < 0.001$

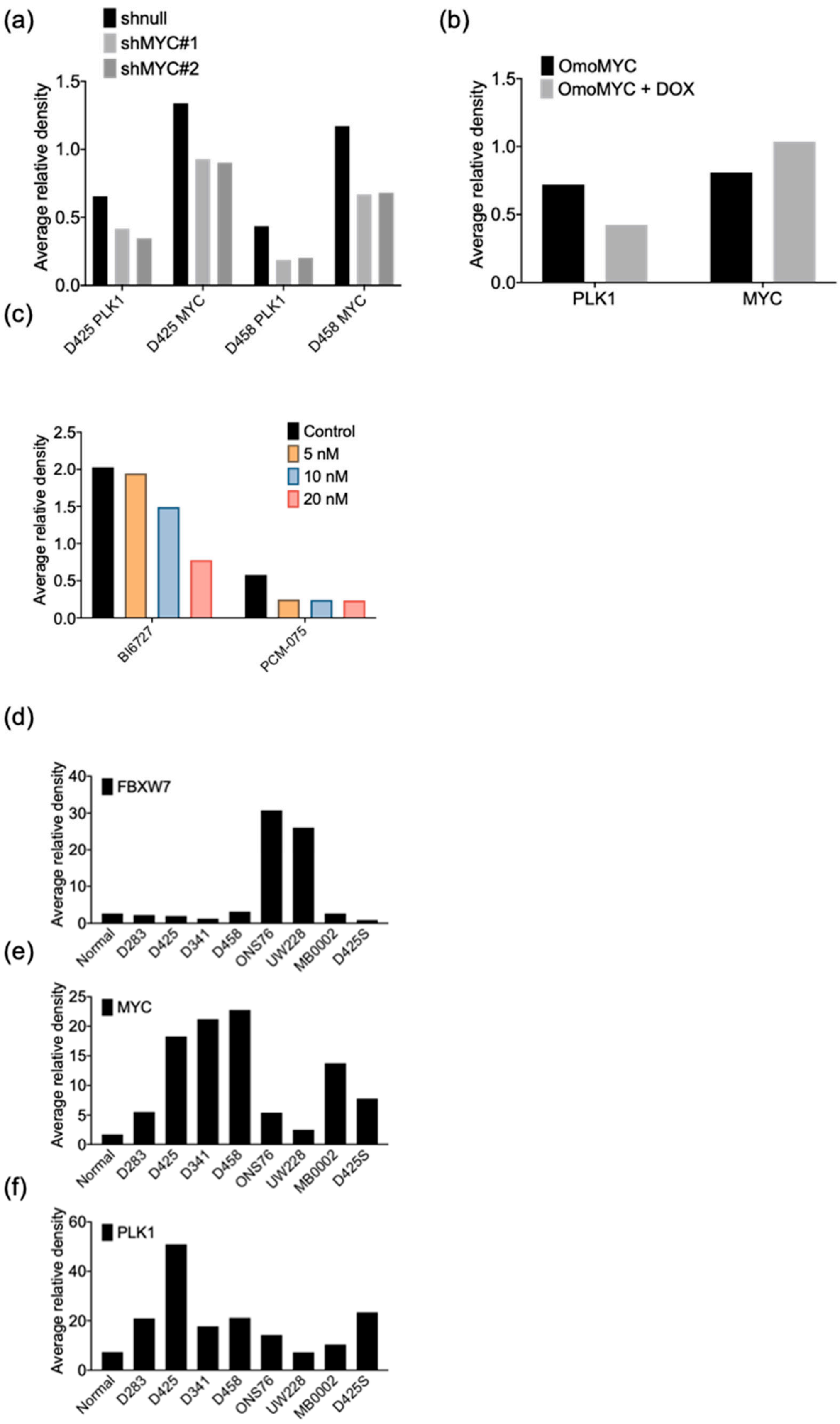

Figure S2. Western blot analysis and quantification. (**a–b**) Western blot analysis and quantification of Figure 1e, 1f. (**c**) Western blot analysis and quantification of Figure 2a. (**d-f**) Western blot analysis and quantification of Figure 3a.

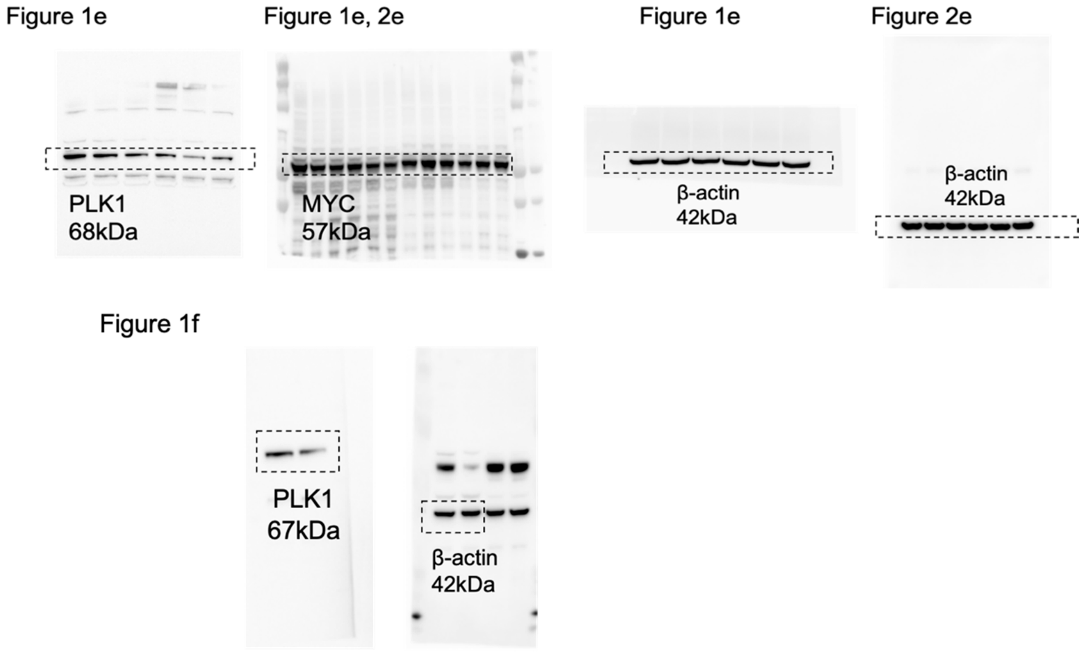

Figure S3. Full blots corresponding to Figure 1 and 2e.

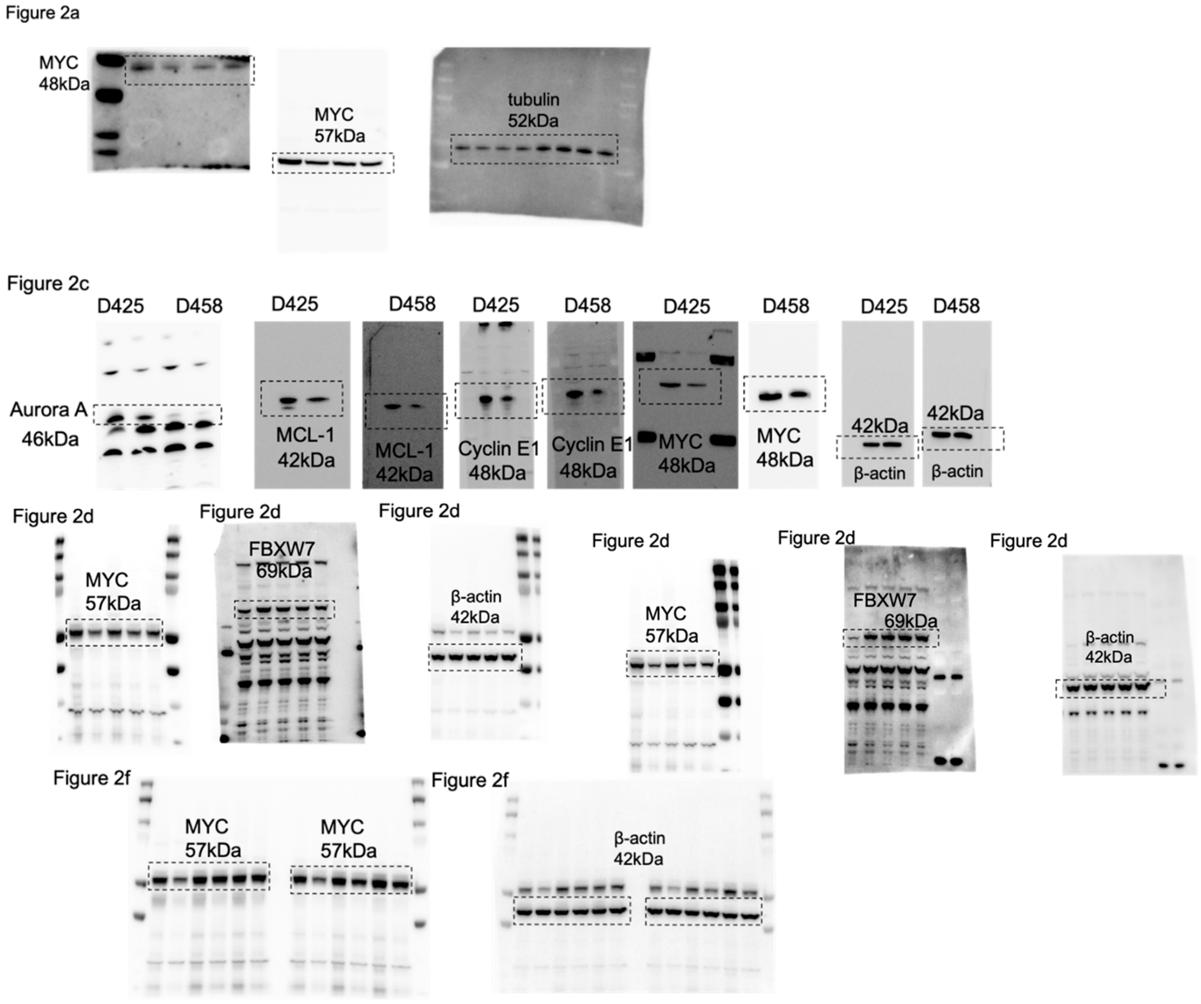

Figure S4. Full blots corresponding to Figure 2.

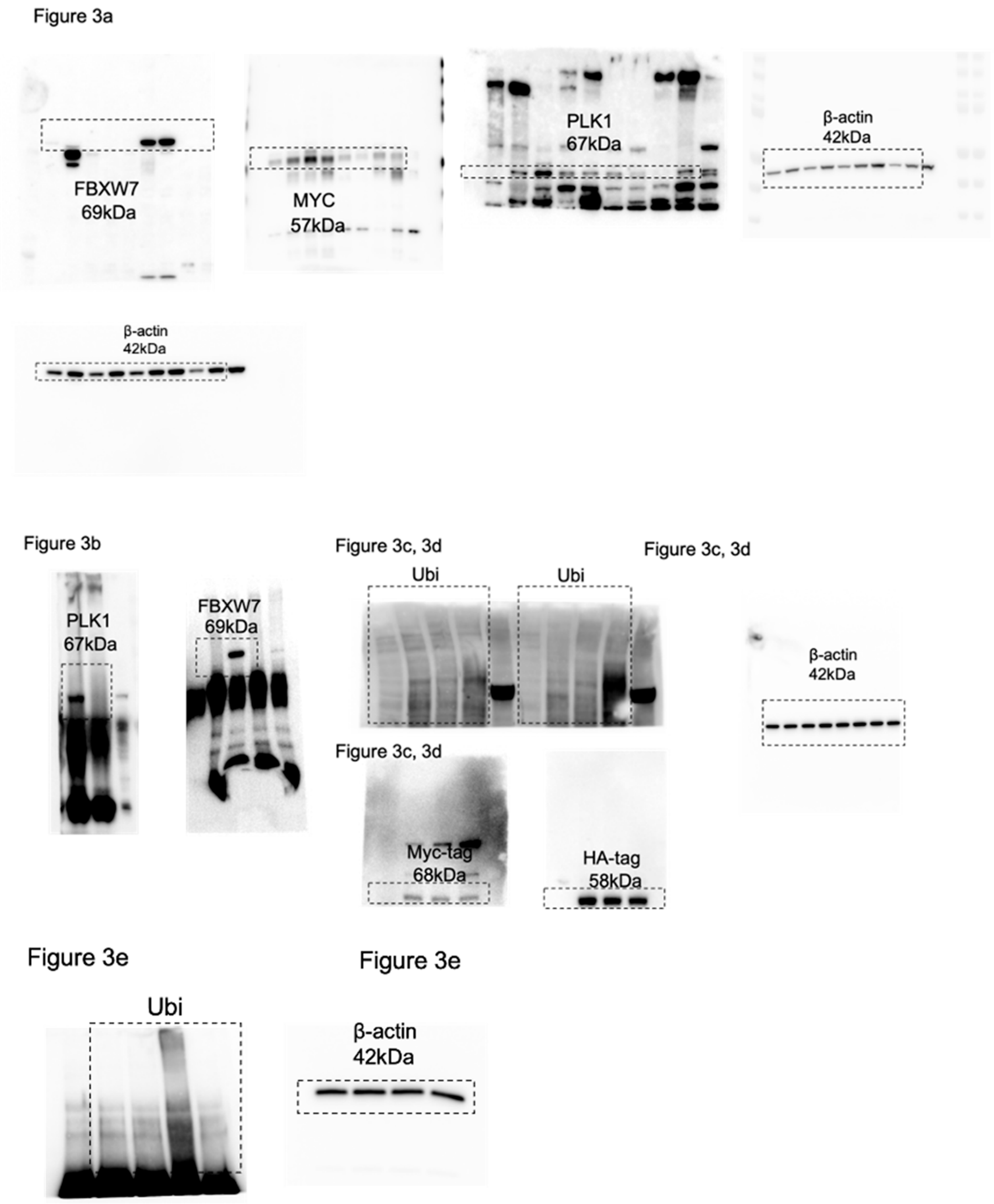

Figure S5. Full blots corresponding to Figure 3.
